# Implementing Competence Committees on a National Scale: Design and Lessons Learned

**DOI:** 10.5334/pme.961

**Published:** 2024-02-06

**Authors:** Anna Oswald, Daniel Dubois, Linda Snell, Robert Anderson, Jolanta Karpinski, Andrew K. Hall, Jason R. Frank, Warren J. Cheung

**Affiliations:** 1 Division of Rheumatology, Department of Medicine, Faculty of Medicine and Dentistry, University of Alberta, Edmonton, AB, Canada; 2 Competency Based Medical Education, University of Alberta, Edmonton, AB, Canada; 3 Royal College of Physicians and Surgeons of Canada, Ottawa, ON, Canada; 4 8-130 Clinical Sciences building, 11350-83 Avenue, Edmonton, AB, Canada; 5 Department of Anesthesiology and Pain Medicine, University of Ottawa, Ottawa, ON, Canada; 6 Institute of Health Sciences Education and Department of Medicine, McGill University, Montreal, QC, Canada; 7 Northern Ontario School of Medicine University, Sudbury, ON, Canada; 8 Department of Medicine, University of Ottawa, Ottawa, ON, Canada; 9 Dept. of Emergency Medicine, University of Ottawa, Canada; 10 Centre for Innovation in Medical Education, Faculty of Medicine, University of Ottawa, Canada; 11 Royal College of Physicians and Surgeons of Canada, 1053 Carling Avenue, Rm F660, Ottawa, Canada

## Abstract

Competence committees (CCs) are a recent innovation to improve assessment decision-making in health professions education. CCs enable a group of trained, dedicated educators to review a portfolio of observations about a learner’s progress toward competence and make systematic assessment decisions. CCs are aligned with competency based medical education (CBME) and programmatic assessment. While there is an emerging literature on CCs, little has been published on their system-wide implementation. National-scale implementation of CCs is complex, owing to the culture change that underlies this shift in assessment paradigm and the logistics and skills needed to enable it. We present the Royal College of Physicians and Surgeons of Canada’s experience implementing a national CC model, the challenges the Royal College faced, and some strategies to address them. With large scale CC implementation, managing the tension between standardization and flexibility is a fundamental issue that needs to be anticipated and addressed, with careful consideration of individual program needs, resources, and engagement of invested groups. If implementation is to take place in a wide variety of contexts, an approach that uses multiple engagement and communication strategies to allow for local adaptations is needed. Large-scale implementation of CCs, like any transformative initiative, does not occur at a single point but is an evolutionary process requiring both upfront resources and ongoing support. As such, it is important to consider embedding a plan for program evaluation at the outset. We hope these shared lessons will be of value to other educators who are considering a large-scale CBME CC implementation.

## Introduction

In an era of greater social accountability, the public has come to expect that postgraduate medical education (PGME) systems have a robust assessment process to ensure the competence of physicians who graduate to unsupervised practice [1]. In PGME, training program directors are responsible for monitoring trainee progress. In the past, they often did so using processes that relied on ad hoc data, supervisors’ remote retrospective impressions, or proxy measures of performance [2,3]. In recent years, postgraduate training has been transformed with the widespread implementation of competency based medical education (CBME); the new model of postgraduate training that seeks to improve the structure of trainee progress by promoting the use of programmatic assessment and group decision-making by a Competence Committee (CC) to guide programs in the systematic collection of trainee performance data for summative assessment of progress [4,5]. While the use of CCs has not been universally adopted in PGME CBME systems, there has been widespread uptake to date in the USA [6,7,8,9,10] and Canada [11,12,13], and growing interest internationally (e.g., Netherlands and Taiwan) [14,15,16].

In programmatic assessment, as trainees progress through their training, they must achieve outcomes of a curriculum described as a series of statements about the expected abilities of graduates. A program of assessment explicitly outlines the assessment strategies and breadth of assessment content and contexts to guide programs in the systematic collection of trainee performance data [17]. Many samples are obtained of a learner’s progress in achieving the desired competencies over the course of the curriculum. Multiple tools and many different assessors provide a variety of inputs into the assessment of trainee progress. While programmatic assessment can be achieved without the implementation of CCs, for example through the program director’s review of the varying assessment inputs, this can be challenging given the volume of data to review and the increased risk of inherent subjective biases with individual reviewers [2,3,18]. Done accurately and effectively, programmatic assessment optimizes learning, facilitates decision making regarding learner progression toward desired outcomes, and informs quality improvement activities of the program [19].

In Competence by Design (CBD), the transformational competency-based change to PGME designed and implemented by the Royal College of Physicians and Surgeons of Canada (hereafter referred to as the Royal College), CCs capitalize on the promissory benefits of programmatic assessment by using quantitative and qualitative assessment data that are collected electronically, curated, and collated into meaningful information describing individual learners and learner populations through learning analytics [20]. Programmatic assessment and CCs have been introduced in a linked fashion in CBD in direct response to calls for improved assessment systems and validity evidence for high stakes summative decisions (e.g., unsupervised practice) [21,22]. Learning analytics and all other forms of assessment data are prepared, reviewed, and synthesized by a trained and dedicated group of educators who comprise the CC to make a collective judgment about progress, promotion, and, ultimately, readiness for unsupervised practice [23]. CC decisions are made collectively, thus incorporating multiple perspectives, to create a broad picture of a trainee’s progression toward competence.

The rationale for CCs draws from the literature on group decision-making, which suggests that groups can reach better decisions than individuals [24] and that systematic group procedures that facilitate greater information sharing can improve group decision quality [25,26]. Thus, CCs may take advantage of group decision-making processes to collectively synthesize and interpret assessment data to make judgments about trainee performance and progress. To maintain fair decision-making within CCs, there should be consistent processes and procedures for how CCs review trainees’ progression. The validity of this summative assessment may be affected by the variability in volume, quality, and interpretation of assessment data within individual programs [27,28,29]. While many benefits of group processes for summative competence decisions have been proposed, CCs are not infallible [26]. There is increasing evidence that lack of member diversity, poor data quality and synthesis, ineffective sharing of information, and groupthink can all threaten the quality and defensibility of group decisions [30,31]. Thus, special attention must be given to these threats in the design and implementation of CCs on a national scale.

In this paper we describe the Royal College model of CCs used in CBD. Here, we reflect on the rationale for the model’s design and desired impact and then outline some of the early successes and challenges that have been noted in the functioning of CCs in Canadian Royal College specialty PGME. Our author group includes physician clinician educators from a variety of specialties. This group includes members involved in the Royal College CC design, implementation and faculty development support and so has detailed awareness of both the aspirations and the challenges through this journey. We recognize that this lens means we bring the biases associated with those who have developed and continue to work to support this national implementation and that this may differ from those who are struggling with rationale or implementation. However, all members of our author group either are or were CC chairs (DD, JK, WC) or CC members (AO, JF, LS, RA) at their local institutions and so we also have lived experience of the CC process on the ground which may help to balance these perspectives.

## The Royal College’s national model of competence committees

While the structure and function of CCs have many similarities across health education systems, the Royal College developed a national model that underpins the work of their CCs in Canada [32]. CCs in the Canadian specialist CBD model of CBME are guided on a national level by processes and procedures recommended by the Royal College in hopes to facilitate optimal group function [33]. The key principles in which the national CC model is grounded are derived from the overarching CBD CBME approach and are outlined in Table 1.

**Table 1:** Key principles of the Royal College of Physicians and Surgeons of Canada’s Competence Committee design.


COMPETENCE COMMITTEE PRINCIPLE	DESCRIPTION

Developmental view of a learner	The system is set up to support progression of competence for all trainees and to ensure that every learner has a pathway to certification. The CC takes a stance that all learners have the potential to be successful, given the right opportunities. The CC supports learners through tailored learning plans paired with guidance on next steps for the trainee’s development toward competence.

Programmatic assessment	The CC uses a comprehensive approach to assessment that capitalizes on multiple data inputs from a variety of data sources and contexts. Data collation and curation allows for an in-depth review of the trainee’s complete portfolio of assessment.

Defined group process with rules	The CC is expected to interpret data in a fair and just manner by applying strategies to mitigate bias and produce defensible group decisions. CC review and deliberation should allow for consideration of diverse views and consensus building. CCs are expected to have common expectations and processes to allow for sufficient consistency within and across programs and institutions.

Criterion-referenced decision-making	The CC is guided by predefined markers of progression, established by the respective national specialty committee, to inform decision-making.

Transparency	The CC is expected to follow structured processes and procedures in their review and deliberation of trainee data to make recommendations of achievement, progress, and promotion. These processes and procedures should be made clear to relevant all invested groups, in particular the trainee.

Clear communication	The CC is expected to use clear communication strategies to ensure the outputs of the committee are communicated to all relevant invested groups


*CC* Competence Committee.

The CC process in CBD is strongly influenced by the structure for CBD curriculum design that was used by every Royal College national specialty committee to create discipline-specific content [34]. The links between the features of CBD and the key principles underlying CC design are outlined in Table 2. In the Royal College CBD model, each national specialty committee took part in a standardized design process (the specialty education design [SED] workshop series) [35]. In this process, the committees set stage-specific expectations of trainee performance and created standards for training and assessment that included guides for the assessment of entrustable professional activities (EPAs), required training experiences, and CanMEDS competencies specific to the discipline [36]. The SED workshops provided an opportunity for the Royal College and front-line clinicians and program directors to engage in co-creation, which maximized the likelihood that local programs would integrate the standards into their CC practices. The CC in each local program uses these national assessment standards to inform their recommendations about trainee progress and promotion. This link to the work of the national specialty committee promotes consistency across diverse local CC contexts, and yet also allows programs to incorporate competencies that are unique to their specific local context. The CC must ensure that both local and national competence expectations are met.

**Table 2:** Links between key CBD design features and Competence Committee principles.


CBD DESIGN FEATURE	COMPETENCE COMMITTEE PRINCIPLES					

	DEVELOPMENTAL VIEW OF TRAINEE	PROGRAMMATIC ASSESSMENT	DEFINED GROUP PROCESS WITH RULES	CRITERION-REFERENCED DECISION-MAKING	TRANSPARENCY	CLEAR COMMUNICATION

**Framework**						

National specialty and stage-specific EPA assessment expectations and required training experiences guide CC decisions	✓	✓		✓	✓	

WBA provides data to support EPA assessment and decision-making		✓		✓		

National accreditation standards ensure minimum requirements of CCs			✓		✓	✓

National guidelines outline common expectations for CC processes and local implementation		✓	✓		✓	

CCs are subcommittees of existing program committee structure					✓	✓

Comprehensive data-informed review values both quantitative and qualitative data sources to inform progress decisions	✓	✓	✓	✓	✓	

Flexibility in educational experiences at local level to ensure achievement of competencies	✓	✓				

**Communication and faculty development**						

National technical guides outline areas where CCs must follow national policy, processes, and accreditation standards and areas of flexibility where local CCs can customize to their settings	✓		✓		✓	

National documents, standards, and expectations are easily accessible				✓	✓	

National, targeted faculty development initiatives support creation and running of CC	✓	✓	✓	✓	✓	✓

**Expanded CC role**						

CC review contributes to the development of individualized learning plans	✓	✓	✓	✓		

CCs are integrated as agents of program, specialty, and CBD CQI nationally			✓		✓	


*CBD* Competence by Design; *CC* Competence Committee; *CQI* continuous quality improvement; *EPA* entrustable professional activity; *WBA* workplace-based assessment.

The Royal College produced documents to guide CC structure and function in the CBD model [32,33]. These documents support consistency in the application of the CC model across all Royal College accredited programs; they include guidance on suitable membership of CCs and CC processes and procedures [33], sample terms of reference for a CC [32], and a technical guide [37] that outlines specific requirements as well as areas of flexibility in CC decision-making. With regard to membership, a minimum of three members is recommended and the committee is encouraged to include a diversity of members (e.g., seniority, gender, urban/rural, physician and non-physician members etc.); this aims to promote diverse interpretation of the data and consideration of differing perspectives [38]. Program directors are encouraged to join CCs as non-voting members to facilitate communication between the CC and the residency program committee (RPC), but they are discouraged from chairing the CC themselves to avoid conflicts of interest and excessive workload [39]. The processes and procedures lay out the expectation that the CC will, in its review, apply a holistic comprehensive approach to a portfolio of assessment beyond clinical competencies [40,41]. The CCs are expected to have access to and incorporate a range of assessment data that may include workplace-based assessments (WBAs) such as EPA observations, non-WBA clinical assessments (e.g., OSCE and simulation assessments), and non-clinical assessments (e.g., research competencies and teaching evaluations). It is expected that the review conducted by the CC will be data driven and grounded in the evidence documented in the trainee’s assessment portfolio. This aims to promote transparency and serves to minimize hearsay or inherent biases [25,42]. To allow for a deep review of the trainees’ portfolios while maintaining efficiency during deliberation at CC meetings, CCs are encouraged to assign a primary reviewer who will complete a deep review of individual files to inform (but not replace) the CC discussion. It is required that programs share CC recommendations with the trainees. The Royal College encourages programs to provide trainees with longitudinal coaches [14,43,44] to help create plans for acting on recommendations made by the CC; however, programs have flexibility to develop other systems that serve the same purpose.

The Royal College CBD model is unique in that CCs make judgments of trainees’ achievement of stage-specific developmentally sequenced EPAs (known as RCEPAs) rather than solely judging their achievement of terminal (end-of-training) competencies. These judgments of competence are categorical achievement decisions (yes/no) for each of the stage-specific EPAs and contribute to the trainee’s progress toward overall competence. In addition, CCs make recommendations to their RPC on the learner’s status (e.g., *progressing as expected, not progressing as expected, accelerated progress*), readiness for progression from one stage to the next, readiness for sitting the certification examination, and, ultimately, readiness for certification [45]. These are meant to be comprehensive decisions, informed by the entire collection of performance data in the portfolio, not only by EPA observation data. In the Royal College CBD model, CCs are tasked with synthesizing assessment data to assign these summative status recommendations within and between stages; they do not determine a “level” or degree of competence along a continuous scale of entrustment for each EPA or milestone as in other CBME models [46,47].

In Canadian PGME, the Royal College is in a unique position to support the national implementation of CCs. Owing to its responsibility for national education design and standard setting, program and institution accreditation, and individual physician credentialing, the Royal College sets standards for multiple aspects of CC functioning. Before CBME, the Royal College had a system in which successful completion of specifically itemized time-based experiences was the basis for decisions regarding examination readiness and credentialing for specialist certification. In CBD, the local CC is responsible for recommending eligibility to sit national specialty examinations and eligibility for certification. However, the Royal College administers these examinations, credentials the candidates (in large part on the basis of the CC’s recommendation), and confers certification. The Royal College develops the national standards for training and assessment applied by CCs and the national expectations for CC functioning, while the responsibility for and oversight of CCs is at the level of each university’s PGME office. This local oversight of CCs is governed by the national system of program accreditation through the Canadian Residency Accreditation Consortium (CanRAC) of which the Royal College is a member; CanRaC includes standards for CCs at both the program and institution PGME level [48,49]. The Royal College CBD CC model recognizes and embraces the notion that responsibility for oversight to deliver graduates who provide safe, high-quality patient care is shared between three entities: the Royal College and its national specialty committees, the local programs and their CCs, and local institutions’ PGME offices.

An important goal of CCs in CBD is to apply a developmental approach that aims to ensure trainees are provided support and guidance to promote further development of their competence and mastery [50,51]. For example, the CC may suggest learning plans or clinical experiences to support success in stage progression or in the national certification examinations. However, this means that the CCs face a dual purpose in that they must take a gatekeeper or public safety role in their determinations of trainees’ progress and assurance of achievement of competence, while also taking a developmental approach in identifying goals and providing direction for further growth.

In the Royal College CBD CC model, CCs have an additional role: the continuous quality improvement (CQI) activities of the discipline-specific specialty committees [31,52]. Specialty committees are asked to review their standards on a regular basis. As part of this review, CCs are invited to identify and report to the national specialty committee, through their program director, instances where revisions may be needed to the national assessment guides or where updated versions of the guides may be required. This provides a link between local CC practices and experiences and national CQI of the specialty education design.

## Challenges and lessons learned

The large-scale implementation of CCs was a complex undertaking, and thus it is not surprising that Royal College educators, program directors, and CC chairs encountered several challenges in the process. Their experiences and the strategies they used to attempt to mitigate those challenges offer lessons learned that may guide others who are planning to implement CCs at scale as they may encounter similar challenges (see Table 3). We do not have all the answers and continue to work with the invested groups in our PGME and Royal College community to co-create solutions as part of this journey. We present what we have learned to date.

**1. Balancing fidelity with flexibility**. The first of these challenges relates to the tension between maintaining consistency and alignment with the key principles of CC implementation and procedures, while also embracing the flexibility needed to accommodate the wide variability between institutions and between programs. Examples of this heterogeneity include the size of the program, the distribution of training sites, the context of the clinical work, the program’s readiness for change to CBME, the sophistication of electronic assessment portfolios, policies at the local institution, the teaching culture, and even fee structures for clinical teachers. For example, small programs may be challenged with a lack of faculty members to achieve a CC quorum or in their ability to minimize conflicts of interests, and larger programs may suffer from having a large number of trainees to review or too many members in the committee to make decisions effectively. Some programs may work closely with their trainees and provide direct observation of clinical work while others may have workflows that rely more heavily on indirect observation for their WBAs. These contextual differences necessitate local adaptations: there cannot be a one-size-fits-all or overly prescriptive approach [5]. To maintain alignment with CC principles in the face of this variability, the Royal College created a national CBME Lead group with representatives from each university and facilitated regular meetings and a national CC community of practice. These two groups have created venues for sharing of best practices and policy management to aid in development and organization of CCs and to promote dialogue between the invested groups as part of a wider communication strategy.

**Table 3:** Challenges and lessons learned in the large-scale implementation of competence committees.


CHALLENGES	ROYAL COLLEGE RESPONSES TO CHALLENGES	INSIGHTS AND LESSONS WE LEARNED ALONG THE WAY

**1. Standardizing process and procedures while maintaining flexibility**	Disseminated national terms of reference and policy documents Articulated where there is flexibility in the process to allow adaptation to local structures and increased ownership through a technical guide Created a community of practice model through the CC chairs forums to help identify and develop best practices among programs with similar contexts Developed annual pulse surveys distributed to invested groups to identify whether processes were implemented as intended and to identify any unforeseen challenges	Provide clear guidance and simplified expectations to ensure consistent messaging and practices Anticipate local adaptations as there is no one-size-fits-all approach Anticipate tensions between flexibility and standardization of interventions Use program evaluation as a key enabler to help identify and mitigate any divergence in practices and to maintain fidelity and integrity during implementation

**2. Addressing the contextual variability within institutions, programs, and systems**	Identified and recruited a national CBME Leads group with Leads within each university Created a network of peers within each university and externally through individual specialties through the CC chairs forums Developed ongoing two-way dialogue between the Royal College and invested groups Organized multiple in-person and virtual CC chairs forums for clear communication, sharing of best practices, and identification of common challenges with implementation	Recognize that each university and individual program will have unique contexts that require adaptable implementation Identify and group common elements related to context (e.g., size of programs, institutional policies, and resources) that can help provide direction on ways to adapt CC implementation Be mindful that when new systems of assessment are applied too rigidly it can lead to frustration or overburdened assessment practices Engage invested groups in the process to create a shared vision and build trust

**3. Working with finite human and financial resources**	Provided centralized investment through development of free key resources (e.g., electronic platform, assessment templates, e-modules, and adaptable slide decks for faculty development) Provided a venue to share best practices and locally developed approaches that could be adapted by institutions via national CBME Leads group and the national CC chairs forum	Recognize and plan to accommodate the wide variations in financial and human resources among institutions and programs Expect the need for and support additional faculty time for portfolio review and attendance at meetings as CCs are a new structure Be mindful that individual institutions may feel more comfortable using existing or locally developed resources, which may increase the resource burden to that institution

**4. Providing faculty development and ensuring engagement**	Developed and maintained a curated repository of online faculty development resources (e-modules, workshops, webinars) Created a national CC chairs forum to enable effective networking, innovation sharing, and movement of knowledge to those who need it to improve their CC practices	Plan for faculty development activities that involve longitudinal and multimodal offerings aimed at all invested groups (e.g., CC chairs, administrators, faculty, and trainees) Develop faculty development strategies that emphasize interconnectedness and relationship building to help support insights on effective knowledge translation in complex systems

**5. Changing the culture of assessment**	Worked toward shared mental models among invested groups of intended CC implementation Ensured alignment of national institutional policies and accreditation standards to avoid confusing or mixed messages	Provide guidance on the policies, processes, and procedures that guide CC functioning Communicate the purpose and flow of CC work to all invested groups to build transparency in the assessment system Acknowledge the dual purpose of assessment for developmental and summative progress purposes while providing rationale and strategies on how to manage this tension Monitor for linear or reductionist approaches to programmatic assessment that can lead to negative assessment behaviors and practices


*CBME* competency based medical education; *CC* Competence Committee.

Standardization of procedures and operations may help provide transparency and consistency in the processes, but there still needs to be flexibility in CC practices to ensure they are focused on the overarching principle of making defensible summative decisions with a holistic view of trainee progress. When processes or procedures are applied too rigidly it can lead to frustration or counterproductive behaviours in CCs, trainees, and front-line faculty [53,54]. CCs may thus focus much of their cognitive efforts on organizing data and reviewing what is easy to collate rather than engaging in reasoning to make sense of all the data. This has led some programs to overvalue quantitative assessments, which can lead to “checkbox behaviours” and a performance orientation or mindset by the learner; or to undervalue these assessments by being overly lenient, which has led to learner or faculty disengagement; or to overly penalize struggling trainees, leading to assessment avoidance [55,56,57]. To enable CCs to function in diverse environments and mitigate some of the challenges outlined above, the Royal College created a national technical guide that explicitly states the standards and minimum requirements and also outlines where there is flexibility [37]. For example, while the intention in the original CBD design was to incorporate the Royal College’s national CanMEDS competency framework into the milestones level, CC review practices were hindered by the limitations of the data reporting features available in the existing electronic portfolios. As such, the technical guide acknowledges these challenges and aims to reduce assessor burden by removing the requirement for milestone assessment scales. The evolution in the Royal College’s approach to CBME as well as the opportunity for contextual modifications while maintaining the principles outlined in Table 1 and articulated in the technical guide have allowed for some flexibility in how CCs function.

Maintaining fidelity of implementation while supporting local adaptations also requires ongoing program evaluation and institutional accreditation to ensure key elements are still met. The program evaluation committee at the Royal College has provided information related to effective CC implementation, and these evaluation reports were disseminated to implementers and key invested groups [57,58]. Given local adaptations, the responsibility for CC oversight, continuous quality improvement, and peer review is shifting to the institution level. This shift to institutionally centred CC oversight is still in the pilot stages of implementation and evaluation. National benchmarking and aggregate data sharing could help to inform the evolution of policies and procedures, but such a step is associated with a high level of complexity and concerns regarding data safety and privacy across institutions.

**2. Resourcing implementation of CCs**. When major curricular changes are planned, there must be sufficient resources to support implementation as well as enough time. Many of the resources required to implement CBD were underestimated or not identified at the outset, which posed significant challenges [59]. Although the true costs of setting up a CC for programs are unknown, consideration needs to be given to several issues: a realistic time commitment from faculty members needs to be established, expenses associated with program administration need to be clearly identified, and the potential for novel assessment data requirements (e.g., multisource feedback, simulation) needs to be examined. Members of the CC should have protected time for faculty development and reviewing activities, which requires support from leadership for financial incentives, time, and/or recognition. Institutions may encounter costs related to support for oversight, program evaluation, and unexpected delays or cost overruns of an electronic portfolio platform. To assist with resource requirements the Royal College provided a centralized electronic portfolio, curated faculty development resources, and undertook a program evaluation for programs requiring this type of support.

**3. Supporting faculty for CCs**. Clear and concise communication of the purpose, process, and procedures for CCs is critical for member engagement and faculty understanding. The Royal College created a plan for communicating a shared mental model among invested groups to develop trust in the system. Importantly, this work supported knowledge dissemination efforts about the underlying principles (see Table 1) to increase the defensibility and acceptability of group decision-making. Throughout the implementation process the Royal College provided multiple longitudinal and reinforcing offerings for trainee and faculty development. These included the development of workshops, as well as a resource directory that included recorded webinars, readily adaptable slide sets, teaching videos, infographics, technical guides, and e-modules [60]. The Royal College also developed a national community of practice model for CC chairs (called the CC chairs forum) to better understand the barriers and facilitators to implementation and to provide ongoing support to CC chairs. An ongoing dialogue and a longitudinal faculty development strategy enabled the alignment of all processes with standard principles and calibrated expectations for CC conduct. Given the high turnover of CC leaders [61], communities of practice require ongoing support and development.

**4. Addressing assessment culture**. The ingrained culture of performance assessment has been the most formidable barrier limiting the integrity of CBME and thus CC implementation. Given their prior experiences and perceptions of assessment, trainees may have a difficult time understanding the dual purpose of assessment as both for and of learning, and they may consider even low-stakes daily assessment as summative [62,63]. This limits the usefulness of feedback provided in WBA as trainees may avoid or negate any feedback that they may perceive as potentially harmful to their progress decisions. Faculty members in the CC may align their roles within a problem identification model and focus all their efforts solely on identifying trainees who are struggling [64]. Although unintentional, these behaviours may limit the underlying benefits of CBME and CCs. The use of CCs to enact programmatic assessment requires careful attention and thoughtfulness when communicating with or developing invested groups. Communicating the nature of the dual purpose of low-stakes observations for high-stakes CC decisions has been challenging both for learners and faculty to adopt or accept, as it requires more complex conversations and fundamental trust in the CC’s goal of taking a developmental view of learner progress. Without these key conversations, there is a risk that a hidden curriculum in which summative components alone are valued will emerge. Those implementing CCs need to work to create a culture of assessment wherein assessment for learning and a growth mindset are emphasized.

## Conclusions

The national implementation of CCs for a new CBME system is complex owing to the expertise and skills required by those delivering and receiving the intervention; the number of groups, settings, and levels targeted; and the permitted level of flexibility of the intervention or its components. With large-scale CC implementation, managing the tension of standardization with flexibility is a fundamental issue that needs to be anticipated and addressed with careful consideration and engagement with invested groups. Anticipating the challenges of implementation in a wide variety of contexts necessitates an approach that uses multiple engagement and communication strategies to allow for local adaptation. Large-scale implementation of CCs does not occur at a single point but is an evolutionary process requiring ongoing support. As such, it is important to consider embedding a program evaluation plan at the outset of implementation. We have presented the Royal College’s CC implementation experience, the challenges that were faced, and some strategies we used to address them. We hope this will be of value to other educators who are considering a large-scale CBME CC implementation.
